# The Clinical Perspective on Hepatitis E

**DOI:** 10.3390/v11070617

**Published:** 2019-07-05

**Authors:** Thomas Horvatits, Julian Schulze zur Wiesch, Marc Lütgehetmann, Ansgar W. Lohse, Sven Pischke

**Affiliations:** 1 Department of Medicine, University Medical Center Hamburg-Eppendorf, 22527 Hamburg, Germany; 2 German Center for Infection Research (DZIF), Hamburg-Lübeck-Borstel and Heidelberg Partner sites, 22527 Hamburg, Germany; 3 Institute of Microbiology, Virology and Hygiene, University Medical Center Hamburg-Eppendorf, 22527 Hamburg, Germany

**Keywords:** Hepatitis E, HEV

## Abstract

Every year, there are an estimated 20 million hepatitis E virus (HEV) infections worldwide, leading to an estimated 3.3 million symptomatic cases of hepatitis E. HEV is largely circulating in the west and is associated with several hepatic and extrahepatic diseases. HEV Genotype 1 and 2 infections are waterborne and causative for epidemics in the tropics, while genotype 3 and 4 infections are zoonotic diseases and are mainly transmitted by ingestion of undercooked pork in industrialized nations. The clinical course of these infections differs: genotype 1 and 2 infection can cause acute illness and can lead to acute liver failure (ALF) or acute on chronic liver failure (ACLF) with a high mortality rate of 20% in pregnant women. In contrast, the majority of HEV GT-3 and -4 infections have a clinically asymptomatic course and only rarely lead to acute on chronic liver failure in elderly or patients with underlying liver disease. Immunosuppressed individuals infected with genotype 3 or 4 may develop chronic hepatitis E, which then can lead to life-threatening cirrhosis. Furthermore, several extra-hepatic manifestations affecting various organs have been associated with ongoing or previous HEV infections but the causal link for many of them still needs to be proven. There is no approved specific therapy for the treatment of acute or chronic HEV GT-3 or -4 infections but off-label use of ribavirin has been demonstrated to be safe and effective in the majority of patients. However, in approximately 15% of chronically HEV infected patients, cure is not possible.

## 1. Introduction

Hepatitis E is infectious disease caused by the hepatitis E virus (HEV), a positive strand RNA virus with worldwide prevalence. The majority of HEV infections are asymptomatic and lead to spontaneous clearance of the virus, while a minority of infected individuals develop clinical overt hepatitis E. Chronic hepatitis E infection can in term then either lead to acute liver failure (ALF) in few patients or to acute-on-chronic liver failure (ACLF) in patients with underlying liver disease. Since the discovery of HEV about 40 years ago, it has been a widespread belief that hepatitis E is a self-limited, tropical disease without large relevance in industrialized countries. However, this view had to be revised within the last two decades: HEV is largely circulating in the western world and is associated with several hepatic as well as extrahepatic diseases [1,2]. HEV has been reported to cause chronic hepatitis E in immunosuppressed patients and a growing number of reported hepatitis E cases from the Western World and the high seroprevalence rates in these countries with improved hygienic conditions demonstrated new, previously unknown facets of this viral disease.

There are eight known HEV genotypes (characteristics of HEV genotypes are illustrated in Table 1). In the tropics HEV genotype 1 and 2 are present and causative for 3 million symptomatic cases, including 56,000 fatal courses, each year [3,4]. These infections occur in the context of epidemic outbreaks and are transmitted by contaminated drinking water. There is no known relevant zoonotic reservoir for HEV genotype 1 and 2. In contrast, single, non-epidemic zoonotic HEV genotype 3 infections occur in Europe, North- and South America and Australia: contact with swine and consumption of inadequately heated pork are sources of infection with Genotype 3. Pork needs to be cooked for more than 5 min at 70 °C to inactivate the HEV virus [5]. Likewise, genotype 4 infections are also transmitted by pork, but this genotype is prevalent in Asia and only single rare cases have been described to be autochthonously acquired in Europe [6]. In addition to pork, fruits and vegetables watered with fecal waters or swine manure infected with HEV, may contain HEV particles on their surface, becoming putative sources of HEV infection [7,8]. Marine filtering animals such as molluscs and seafood, can concentrate HEV particles and be a potential source of HEV transmission [9,10,11,12,13]. Furthermore, the relevance of HEV infection, transmitted by blood products has been discussed controversially within the last decade [14,15,16,17]. Approximately up to 1/1000 blood donations in Europe tests positive for HEV, thus several European countries decided to test blood donations regularly for HEV to avoid blood borne HEV transmission to weakened patients in need of receiving blood products [14,18]. Transfusion of blood products (red blood cells) containing HEV RNA can cause acute hepatitis E in an immunocompetent patients as recently reported [19]. Interestingly, Riveiro-Barciela et al. recently reported a case of HEV associated exacerbation of thrombotic thrombocytopenic purpura transmitted by cryosupernatant plasma [20].

**Figure 1:**
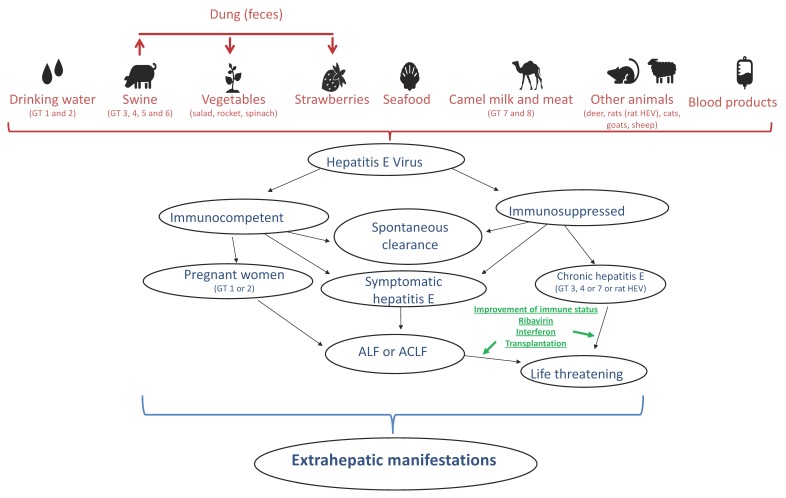
Different sources and routes of infection and course of disease in immunocompetent and immunosuppressed individuals.

Recently, HEV genotypes 5 to 8 have been identified. HEV 5 and 6 have been isolated from wild boars, while HEV 7 and 8 have been found in dromedary and Bactrian camels, respectively [21,22]. Single cases of human infections with these genotypes have been described, but their epidemiological relevance for human medicine is still unknown. Furthermore, single cases of rat HEV to humans have been reported. Rat hepatitis E differs largely from the eight known humanopathogenic genotypes and has been assumed not to be able to infect humans. Recently rat HEV has been detected for the first time in Great Britain [23]. However, this distinct HEV species seems to be able to infect humans and even cause chronic hepatitis E in immunosuppressed patients as recently reported from Hong Kong [24]. Very recently the first case of rat hepatitis associated acute hepatitis in an immunocompetent patient in Canada was reported [25]. Surely the coming years will clarify the role of genotype 8. Different sources and routes of infection and course of disease are illustrated in Figure 1.

**Figure 2:**
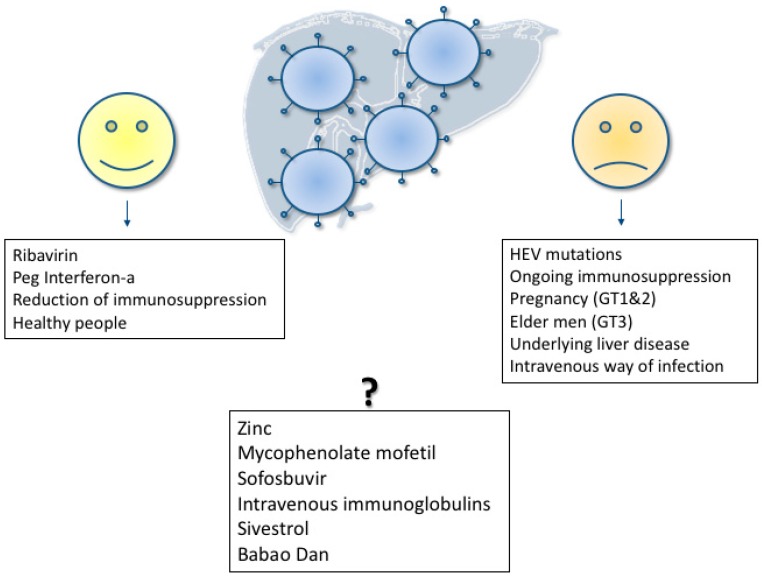
Different factors influencing HEV infection: Protective and beneficial factors on the left hand; unfavorable and risk factors on the right hand; not yet clarified factors in the lower middle.

Anti-HEV IgG seroprevalence rates of up to 30% in Germany and even higher in France and the Netherlands have been reported [26]. A recent meta-analysis revealed that the risk of HEV exposure in the United States of America is nearly double as high in comparison to the neighboring Latin American countries [27]. In immunocompetent individuals, the great majority of infections with HEV leads to a clinically silent disease course. It is still unknown how many of the exposed individuals seroconvert to anti HEV and how many infected individuals never develop clinical signs of HEV infection. However, many individuals seroconvert without any symptoms or laboratory signs of hepatitis, such as increased alanine aminotransferase (ALT) or aspartate aminotransferase (AST) values. In contrast to these asymptomatic HEV infections, a minority of patients develops an increase of transaminases caused by viral hepatitis. Usually the ALT is higher than the AST in these patients. Based on data generated with blood donors it has been extrapolated that approximately 10% of these patients develop symptoms of hepatitis, such as fatigue, jaundice or weakness [18]. Particular risk groups for a severe HEV course are pregnant women infected with genotype 1 (but also other genotypes) and elderly men or patients with underlying chronic diseases for genotype 3. In addition to the liver related clinical manifestations of hepatitis E, HEV has been associated with several extrahepatic manifestations. Even if this association is strong in some of these extrahepatic symptoms, a causal pathophysiological link has still not been proven. Different factors influencing HEV infection are shown in Figure 2.

A stem cell derived cell culture system has been established to study the in vitro replication of HEV and possible inhibitors [28]. Furthermore, human liver chimeric mice have been established as a model of chronic hepatitis E virus infection for preclinical drug evaluation [29,30]. In addition, there is increasing evidence that HEV-specific T cell responses contribute to the control of HEV infection [31]. Recently, HEV-specific T cell responses have been characterized targeting the entire HEV genome without distinct immunodominant regions [32]. However, a possible link between a T-cell response against distinct HEV epitopes and the clinical course of HEV infection has still not been proven.

Therapeutic options for chronic hepatitis E include reduction of immunosuppression in order to improve the immune status of the patient [33], therapy with interferon α [34,35] or treatment with ribavirin [36,37,38,39]. In 2012 a recombinant HEV vaccine was approved for use in China. This vaccine showed an efficacy of >90% in preventing acute symptomatic hepatitis E [40]. However, this vaccine is effective against HEV genotypes 1 and 4. It is still unknown if it is also protective from HEV genotype 3 infections. In 2018 the European Association for the Study of the Liver (EASL) released their clinical practice guidelines on hepatitis E [41].

## 2. Acute Hepatitis E

### 2.1. Autochthonous HEV Infections in Immunocompetent Individuals in Industrialized Countries

In contrast to the tropical variants (genotype 1 and 2), the HEV infections in industrialized Countries (caused by genotype 3 and 4) seem to take a less severe course of disease [42]. However, HEV genotype 3 infections have been described to induce acute or acute-on-chronic liver failure in elderly men or patients with underlying liver diseases. The potential differential diagnosis of acute HEV infection should be ruled out in patients with suspected drug induced liver injury (DILI) or first appearance of autoimmune hepatitis [43,44,45,46,47]. Surely the role of HEV infection as potential trigger of acute-on-chronic liver failure is of major interest.

### 2.2. Hepatitis E as Cause of ACLF

HEV infection may result in ACLF in patients with underlying liver disease. Previous studies have shown that HEV infections increase the risk of death up to 70% in cases of ACLF [48,49]. Typical features of ACLF include acute deterioration of liver function with clinical complications of hepatic decompensation such as onset or deterioration of ascites, hepatic encephalopathy and/or hepatic coagulopathy. ACLF is associated with dramatically increased mortality rates [50,51]. A large British/French study on 343 patients with decompensated liver disease identified 3% of decompensations to be associated with acute HEV infection (*n* = 11), three of these patients died [52]. Another study investigating 368 cases of ACLF in India reported an incidence of 12% of HEV associated infections. However, mortality in HEV-ACLF was lower at 18% compared to other ACLF etiologies [53,54]. In contrast, in West Africa acute HEV infection was not observed as a common cause of ACLF [55].

Cases of HEV induced ACLF in patients with underlying liver diseases seem to be similar to the hepatitis A virus (HAV) induced cases of ACLF. Particularly, patients with chronic hepatitis C virus infection but not with chronic hepatitis B virus infection are at risk for decompensation under the condition of HAV superinfection [56]. Such an association has not been reported for hepatitis E.

While tropical HEV genotype 1 and 2 infections sometimes result in acute liver failure in patients without any underlying diseases, development of ALF in healthy people with HEV genotype 3 or 4 infections presents a rarity [57].

### 2.3. Hepatitis E in the Tropics

HEV genotype 1 is the predominant genotype for endemic and epidemic HEV-infections in Asia and Africa, whereas HEV genotype 2 is endemic in Western Africa and regions in Latin America, such as Mexico. Route of transmission of these genotypes is mainly fecal-orally due to contaminated water and reduced hygienic conditions. A single study, by Caron et al., reported the possibility of HEV genotype 1 infecting swine [58]. However, zoonotic transmission of this genotype has not been confirmed by any other studies and does not play any role.

Generally the incubation period of Hepatitis E has been suggested to range between three and eight weeks [3]. A recent study investigating travel-associated HEV genotype 1 infections reported an average incubation period of median 30 days [59]. Typical symptoms in acute hepatitis E are (comparable to other viral hepatitis) unspecific weakness, elevated body temperature, arthralgia or nausea [41]. Some patients with acute Hepatitis E develop jaundice, with itching and typical clinical signs as light-colored stool and darkened urine. These clinical symptoms usually begin shortly after an elevation of aminotransferase levels (ALT > AST). Elevated ALT levels are frequently accompanied by altered cholestasis parameters, such as bilirubin, alkaline phosphatase and gamma-glutamyl transferase [3]. Viremia usually peaks during the early symptomatic phase and becomes undetectable about two weeks thereafter, whereas excretion of the virus in the feces remains 2–3 weeks longer [60,61]. Occurrence of acute liver failure is a rare observation. However, in particular pregnant woman exposed to HEV genotype 1 are at elevated risk to develop fulminant liver failure with a fatality rate of approximately 20%.

In contrast to HEV genotype 3 and 4 infections, chronic HEV genotype 1 or 2 infections have not been described. Furthermore, three studies from genotype 1 regions searched systematically for chronic courses in immunosuppressed individuals but no chronic case was observed: Naik et al. studied a cohort of 205 kidney transplant recipients from India. Twenty-two percent of them had elevated liver enzymes but no case of persistent HEV infection could be verified by PCR [62]. In addition, Feldt et al. studied a cohort of 1544 HIV infected patients from Ghana and Cameroon but also in this study no chronic case was found [63]. Finally, Agarwala et al. followed 30 liver transplant recipients in an HEV hyperendemic region in India. Despite six of them (20%) demonstrating serological signs of recent HEV infection within the six months post-transplant, none of them developed chronic HEV infection [64]. Taken together, these findings rule out the possibility of HEV genotype 1 infections evolving to chronic infections. In contrast to these findings, Robbins et al. reported a single case of chronic HEV genotype 1 infection in a patient with inflammatory bowel and steroid medication disease and HEV genotype 1 [65]. However, this case report has several limitations and thus it should not be overrated [66].

### 2.4. Hepatitis E during Pregnancy

The role of HEV in pregnant women in the tropics is of central importance. It is known that HEV genotype 1 infection during pregnancy in tropical countries (such as India or others) is associated with high maternal morbidity and mortality (with mortality rates up to 20%) [67]. Recently, also HEV genotype 4 infection has been reported to be associated with preterm birth and abortion [68]. HEV infections are associated to eclampsia gravidarum, hemorrhagic complications and liver failure [3,67,69]. Patients may present with nausea or vomiting and mild to moderate rise of aminotransferase levels. However, pregnant women are at risk to develop acute liver failure defined as onset of jaundice, hepatic coagulopathy and hepatic encephalopathy. Both maternal and fetal death but also abortion or premature delivery are complications of Hepatitis E during pregnancy. Overall, HEV infections cause up to 70,000 deaths and 3000 stillbirths per year in tropical regions [4].

The causal mechanisms of fatal courses in pregnancy are still not fully understood. However, hormonal and immunological factors but also genetic factors seem to be of relevance. Steroid hormones which are elevated during pregnancy are known for their immunosuppressive properties and have been reported to enhance viral replication [67,70,71]. Bose et al. suggested a link between diminished expression of the progesterone receptor and fatal outcome of hepatitis E in pregnant women [72]. Furthermore, recently estrogen and its receptors ESR1α and ESR2β have been reported as potential biomarkers predicting worse fetal and maternal outcome in pregnant women with HEV infection [73]. It was furthermore shown that HEV replication occurs in human placenta, and is associated with fetal and maternal mortality in patients with ALF [74]. In summary, multiple hormonal factors seem to influence the clinical course and outcome of HEV infections and thus further studies are needed, investigating the role of genetic HEV variants, hormonal and immunological factors as well as genetic host factors [71].

Unfortunately, there is no specific therapy for pregnant women with HEV induced acute liver failure. Some Indian centers try to treat the inflammation with steroids, but mainly supportive treatment is used. In some cases, liver transplantation presents the only possible causative option [75]. However, to prevent severe HEV infection in pregnant women, a large vaccine trial is currently studying the Chinese HEV vaccine (Hecolin) in more than 20,000 women in Bangladesh (clinicaltrials.gov, NCT02759991).

In contrast to the tropics there are no reports on frequent occurrence of liver failure in pregnant women with HEV infection from genotype 3 or 4 regions, indicating that liver failure in pregnant women is a special feature of tropical HEV infections (GT 1 and 2). Some single case reports indicate that under special circumstances HEV genotype 3 infections in Europe may also take a clinical overt course [76,77]. However, these single patients cleared the HEV infection rapidly without any signs of severe courses.

### 2.5. Acute and Chronic HEV Infections in Organ Transplant Recipients

HEV infections in immunosuppressed individuals can cause chronic courses with possibly development of life-threatening cirrhosis. Especially solid organ transplant recipients are of relevance in this context [78]. Gerolami et al. and Kamar et al. described in the year 2008 for the first time chronic courses of HEV infections in liver and kidney transplant recipients [79,80]. Additionally, cases of chronic HEV infection have been reported in lung transplant recipients and heart transplant recipients [81,82]. While there are several possible differential diagnoses for liver transplant recipients with elevated transaminases (rejection, recurrence of underlying disease, CMV, EBV, other viral infections, de novo autoimmune hepatitis, etc.) there are less possible causes of liver dysfunction in kidney, heart or lung transplant recipients. Thus, hepatitis E is of special relevance as differential diagnosis in these patient cohorts.

Chronic hepatitis E leads to structural changes in the liver, including histopathological alterations such as nodules and fibrous septa, fibrotic remodeling and subsequent cirrhosis [80,83]. Chronic hepatitis E has been reported for GT3 and GT4 infections, while no cases of chronic HEV infections for GT1 or GT2 have been described [62,84,85]. High rates of 47% of chronification of hepatitis E in French kidney transplant recipients with overt hepatitis E have been reported [86]. In contrast, in a cohort of German heart transplant recipients only 4/19 (21%) patients with HEV infection after transplantation developed chronic hepatitis, but this observation does not only base on patients with overt hepatitis E but also those with clinical silent seroconversion [81]. It can be summarized that approximately 20–50% of transplant recipients getting in contact with HEV develop chronic infection.

How chronic HEV infection should be defined has been debated for a long time. In 2013 Kamar et al. contributed largely to this discussion [87]. In a cohort of 69 solid organ transplant recipients with HEV infection, 41 (59%) developed persistence of HEV for more than six months and 28 (41%) cleared the infection within three months. However, no patient spontaneously cleared the infection in more than three but less than six months. Thus, the authors concluded to define three months as cut-off for chronicity in solid organ transplant recipients. However, it could be demonstrated that spontaneous clearance of HEV infection in transplant recipients is possible in some patients more than three months after first detection of HEV viremia [88]. Furthermore, some immunocompetent, healthy individuals can be HEV-carriers for more than six months [18].

## 3. Hepatitis E in HIV Infected Patients

Since 2009 cases of chronic hepatitis E in patients with underlying HIV infection have been described repeatedly [78,89,90]. Interestingly, HEV infection has been described to persist in patients with a strongly impaired CD4 T cell count despite successful suppression under antiretroviral therapy. Thus, an HEV infection previously established under strong immunosuppression can persist despite the immune system having recovered [83,91].

## 4. Chronic Hepatitis E beyond Transplant Recipients or HIV Infected Patients

In addition to transplant recipients or HIV-positive patients, chronic HEV infections in patients with different underlying medical conditions that require immunosuppression including rheumatological and hematological diseases have been reported.

In a multicentric European cohort of 21 rheumatological/internal medicine patients with various disturbances of their immune system, seven (33%) developed chronic HEV infection persisting for more than three months [92]. Two of these patients had been treated with high doses of monotherapy with steroids, one with combination therapy of steroids and mycophenolate and one with steroids and sirolimus. These data demonstrate that chronic hepatitis E is not limited to transplant recipients or HEV patients but also occurs in rheumatological patients with less strict immunosuppression. Ribavirin could be used safely and effectively in these patients to clear the infection.

Furthermore, a potential association of HEV and lymphoproliferative disorders (LPD) has been reported [93,94,95]. Mallet et al. detected HEV in the dermal endothelium being associated with induction of primary cutaneous CD30(+) T cell LPD. The authors conclude that antiviral therapy should be considered in treatment of T cell LPD involved in the skin [93].

Another group of patients at risk to develop chronic hepatitis E are hematological patients, particularly stem cell transplant recipients. Versluis et al. screened a cohort of 328 stem cell transplant recipients for HEV RNA and anti HEV antibodies. HEV RNA was tested by PCR in episodes of elevated transaminases [96]. The authors identified eight cases of HEV infection (2%), five of them developed chronic hepatitis E. Four of the HEV infected patients died from various critical complications with ongoing inflammation of the liver, while the four remaining patients survived and cleared the infection. It remains speculative how much HEV infection contributed to the development of the fatal courses. However, recently a multicentric European study [97] summarized the clinical courses of 50 hematological patients, including 21 (42%) stem cell transplant recipients. Death with ongoing hepatitis E occurred in eight (16%) patients, including one with non-Hodgkin lymphoma (NHL) and one >100 days after alloHSCT in complete remission. Male sex and presence of cirrhosis were associated with fatal courses (*p* = 0.04, *p* = 0.006). Ribavirin was administered to 24 (48%) patients with a sustained virological response of 79.2%, and associated with lower mortality when started <24 weeks of hepatitis E diagnosis (14.3% vs. 66.7%, *p* = 0.037). This study indicates that HEV infection in these really special, weakened, hematological patients might present an additional hit and might lead to death of patients in combination with the crucial underlying diseases. Furthermore, physicians, awaiting an HEV flare triggered by chemotherapy, might decide to delay the required oncological treatment of underlying diseases until HEV infection has been cleared. Thus, it can be summarized that it is reasonable to start ribavirin therapy in hematological patients early and not to delay required chemotherapy/stem cell transplantation.

## 5. Extrahepatic Manifestations of Hepatitis E

In addition to the inflammation of the liver, hepatitis, several other symptoms have been assumed to be extrahepatic manifestations of acute or chronic or previous HEV infections [2]. While pancreatitis has been frequently observed in HEV genotype 1 infections, neurological, hematological, immunological and renal diseases have been frequently observed in patients with HEV genotype 3 infections.

Within the last five years especially, the neurological symptoms have got a lot of attention. In 2017 a multicenter study involving Great Britain, France and the Netherlands studied 464 patients presenting with various non-traumatic neurological symptoms. Surprisingly 2% of them suffered from ongoing (PCR positive) or recently cleared (PCR negative but IgM positive) HEV infection [98]. Especially the association of HEV and neuralgic amyotrophy seems to be proven. Neuralgic amyotrophy is a peripheral nervous disease affecting the plexus brachiocephalicus. Patients suffer from pain, weakness and hypesthesia in one or both shoulders. These symptoms can potentially persist for several months. A large multicenter study investigated 57 patients with this rare disease and simultaneous HEV infection and compared the patients’ characteristics with 61 neuralgic amyotrophy cases without HEV infection [99]. Patients with HEV infection showed significantly more frequently bilateral involvement, damage outside the brachial plexus and involvement of phrenic nerve and lumbosacral plexus injury [99].

Additionally, a study from China demonstrated 5% of Myasthenia patients (*n* = 188) to be anti HEV IgM positive and 2% to be viremic [100]. Furthermore, Guillaine Barre syndrome, an inflammatory neuronal disease, affecting peripheral nerves has been associated with several infectious diseases in general and HEV infection in special: van den Berg et al. compared 201 GBS patients and 201 healthy controls [101]. Anti HEV IgM positivity was observed in 10 HEV positive and 1 HEV negative patients (5% vs 0.5%, odds ratio 10.5, 95% confidence interval 1.3–82.6, *p* = 0.026). Furthermore, meningitis and encephalitis have been observed in some cases of acute or chronic HEV infection and HEV replication could be proven in neurological tissue. Thus, the association of HEV-infections and various neurological diseases seems to be likely, despite the pathophysiological mechanisms of these extrahepatic manifestations not being fully understood.

In contrast to these observations, a large Chinese study investigating 1117 patients with various neurological diseases and 1475 healthy controls from an HEV GT 4 endemic area. In this study anti HEV IgM positivity could be observed in 0.54% (*n* = 6) of neurological patients and 0.68% (*n* = 10) of healthy controls [102]. All anti HEV IgM positive patients tested PCR negative. Thus, the association of HEV and neurological disorders seems to be a feature of HEV GT 3 but not GT 4 infections and this finding seems to present a further differentiation between these two genotypes [103].

In addition to the neurological diseases, various other diseases have been assumed to present possible extrahepatic manifestations of acute or chronic HEV infections. Cases of pancreatitis have frequently been observed in HEV genotype 1 infections but not in genotype 3 or 4 infections [2]. Single case reports have described cases of thyroiditis, myocarditis and hematological disorders in the context of acute hepatitis E. However, data from larger cohorts or studies are available that analyzed frequency and risk factors of these complications and thus these associations are far from clear [2]. The possible association between HEV infections and immunological diseases has been discussed frequently in recent years. Cryoglobulinemia, glomerulonephritis and autoimmune hepatitis have been observed in patients with acute or chronic HEV infection or previous HEV exposure, anti HEV IgG positivity [2]. It still needs to be clarified if this assumed association is founded in heterologous immunity of HEV and an overwhelming immune response triggered by HEV or if HEV replication in various extrahepatic tissues plays a role or if these diseases are not really linked to HEV by a causal relationship. Prospective studies and registers are needed to confirm the association between HEV and these diseases.

HEV infection has also been associated with manifestation in the kidney, as HEV-Ag was detected in kidneys of Mongolian gerbils which were infected with swine HEV [104]. Furthermore, both HEV-Ag as well as HEV RNA could be detected in the urine of a chronically HEV infected patient [105]. Del Bello et al. observed a case of membranoproliferative glomerulonephritis in a kidney transplant recipient with chronic HEV infection, that was treated with ribavirin successfully [106].

An intriguing observation regarding HEV has recently been made by a Chinese group. Huang et al. described a surprisingly high rate of HEV (genotype 4) positivity of 28% in semen of infertile men (*n* = 185) [107]. Furthermore, the authors were able to experimentally infect macaques and detect HEV immunohistochemically in testes of these monkeys. These data strongly suggest HEV to play a role for the development of male infertility in China. In contrast to this observation, a German study did not observe any HEV positive result testing semen of 87 men from an infertility outpatient clinic [108]. Thus, there is no evidence for an association of male infertility and HEV genotype 3 infections. Eventually these two publications might highlight another remarkable difference between HEV genotype 3 and 4 infections.

## 6. Treatment of Acute Hepatitis E

There are no HEV specific drugs approved for the use in HEV infected patients. Thus, all medicinal treatment approaches present off-label use.

In the vast majority of cases, acute hepatitis E is a self-limited, innocuous disease that does not require any treatment. However, in genotype 1 or 2 infections acute liver failure (ALF) might develop especially in pregnant women. In non-pregnant patients with HEV genotype 1 induced ALF or ACLF, ribavirin has been used in five patients in total [38,109]. No severe adverse effects occurred in these patients and all survived. However, systematic double-blind placebo controlled data in this context are still missing. The situation in pregnant women with HEV genotype 1 or 2 induced ALF is much more serious. According to the approval, Ribavirin is contraindicated in pregnant women, as teratogenic effects have been observed for this drug. Currently no case report or study has been published describing the use of ribavirin in pregnant women with HEV induced ALF. However, ribavirin has been approved for the use in HCV infections and despite the contraindication in the case of pregnancy there are some single pregnant women who have been treated with ribavirin. Two hundred and seventy two cases of pregnant women treated with ribavirin directly prior or during pregnancy or with partners taking ribavirin currently or in the past six months have been enrolled in a large pregnancy registry [110]. Based on these data, the authors did not observe a clear signal of human teratogenicity for ribavirin. It should be mentioned that ALF in the context of HEV and pregnancy usually occurs in the third trimester, at this late-term time point the fetal development has a finished organogenesis. Furthermore, it should be kept in mind the acute hepatitis E caused by genotype 1 or 2 has a mortality of 20% in pregnant women. Thus, it should be reflected to use ribavirin in this context, but currently there are no known approaches to initiate a clinical trial regarding to this topic.

**Figure 3:**
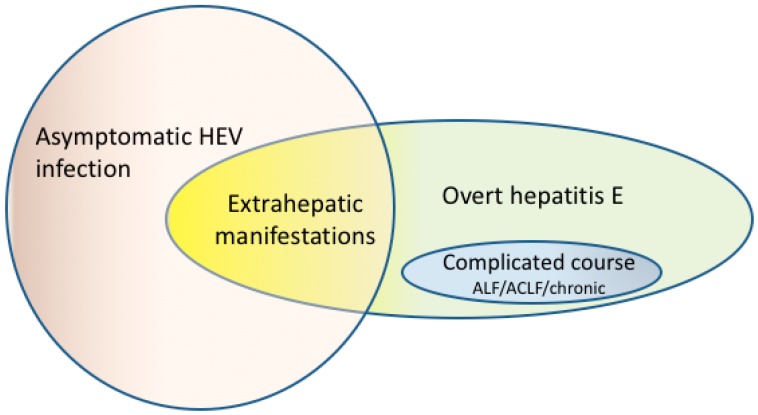
Schematic representation of HEV infection: In the vast majority, patients have a clinically asymptomatic course. Acute hepatitis E is generally self-limited. A minority of infected individuals develop clinically overt hepatitis E. In rare cases HEV infection can result in acute liver failure (ALF) or in acute-on-chronic liver failure (ACLF) in patients with preexisting liver disease. Chronic hepatitis E can occur in immunosuppressed patients.

In genotype 3 or 4 infections there are only very rarely situations to decide to treat with ribavirin. The majority of autochthonous HEV infections in Europe are absolutely asymptomatic and symptomatic cases of hepatitis E caused by genotype 3 usually take milder courses in comparison to imported genotype 1 cases [38,42]. Thus, in the great majority of acute HEV infections in Europe there is no reason to consider treatment with ribavirin. However, HEV genotype 3 can cause acute or chronic liver failure in patients with underlying liver diseases or fulminant hepatitis in elderly men. In the rare situation of ALF or ACLF caused by HEV genotype 3, ribavirin has been shown to be safe and effective in single cases [111,112]. In a multicenter retrospective analysis of nine cases of acute HEV infections treated with ribavirin (600–800 mg/d, for a median duration of 26 days) all patients cleared the infection and no fatal case could be observed [113]. However, large placebo controlled prospective studies are still missing. A schematic representation of types of HEV infection is given in Figure 3.

## 7. Treatment of Chronic Hepatitis E

Approximately 20–50% of HEV infections in transplant recipients progress into chronic hepatitis E. Chronic hepatitis E can lead to cirrhosis in approximately five years. In some immunosuppressed patients with HEV infections it is possible to reduce the immunosuppression, for example, sometimes it is possible to reduce immunosuppressive medication in liver transplant recipients or to initiate antiretroviral therapy in HIV infected patients. In contrast to these groups, in heart, lung or kidney transplant recipients a reduction of the medicinal immunosuppression is in the majority of cases not riskless feasible.

The EASL Clinical Practice Guidelines recommend firstly to reduce the immunosuppression, if possible and thereafter to start ribavirin treatment [41]. Reduction of immunosuppression in 16 solid organ transplant recipients with chronic hepatitis E led to clearance of HEV in four cases (25%) [114].

Ribavirin presents the next step suggested by the EASL guidelines [41]. However, approximately 5–15% of transplant recipients will not clear the infection despite ribavirin treatment (first-line ribavirin response: 78%) [39]. In liver transplant recipients, PEG interferon α could be an alternative, but in heart or lung transplant recipients this is contraindicated due to the high risk of possible graft rejection [41]. In summary, ribavirin presents the most frequently used therapeutic option at many transplant centers. However, ribavirin treatment failure occurs in 15% of transplant recipients. Various mutations in the HEV genome have been associated with the clinical severity of the infection and in particular with the response to ribavirin [115]. The most popular of these genetic HEV variants present the HEV polymerase variant G1634R. This has been associated with treatment failure and has been demonstrated to have increased replication fitness in vitro [116]. The exact relevance of the G1634 variant for treatment response requires further investigation as some patients did not clear HEV infection under ribavirin treatment despite absence of this mutation. The G1634R variant has also been present as part of a minor viral population already, before ribavirin treatment in patients with subsequent treatment failure [117]. It can be concluded ribavirin induces HEV mutagenesis selection of viral strains with an improved fitness, these HEV variants may emerge during treatment [117,118].

The need of an adequate and successful therapy for the 15% of chronically HEV infected patients with ribavirin treatment failure led to the evaluation of alternative treatment strategies. Reduction of immunosuppression and interferon are harboring the risk of rejection and contraindicated in many patients as lung or heart transplant recipients without the option of bridging therapy in the case of severe rejection.

There are some case reports and in vitro data displaying the potential use of sofosbuvir in this context. Sofosbuvir, a NS5B polymerase inhibitor, has been developed for the treatment of hepatitis C and presented the first interferon free treatment option for this disease. Sofosbuvir was reported to have an antiviral activity against HEV in vitro [119]. van der Valk et al. observed a decline in HEV RNA in a patient who failed to clear HEV with ribavirin therapy receiving sofosbuvir. However, this and other patients experienced viral relapse after the end of therapy or failed to clear the infection [120,121]. A recent, small study has investigated the effect of sofosbuvir monotherapy in 10 chronically HEV infected patients, who failed to achieve HEV clearance under ribavirin therapy or who presented contraindications against ribavirin treatment. While the viral load initially significantly decreased in many of these patients, none of them reached an SVR [122].

Additionally, in vitro studies demonstrated that silvestrol, a natural compound isolated from the plant Aglaia foveolata and Babo Dan, a Chinese herb, show antiviral HEV effects in vitro and in vivo (rabbits) [123,124]. The clinical relevance and potential use of these substances still needs to be defined in controlled studies. Furthermore, a potential anti-HEV effect of Zinc supplementation has been suggested basing on in vitro data and two patients who experienced treatment failure under ribavirin monotherapy but cleared the infection under Zinc/ribavirin combination [125] but further studies are needed to uncover the pathophysiological aspects and the potential clinical role of Zinc in chronically HEV infected patients with ribavirin treatment failure.

## 8. Vaccine

After several attempts to develop a vaccine against hepatitis E, in 2007 a successful phase II trial of a novel HEV vaccine was described [126]. This vaccine based on a protein encoded by ORF2 of an HEV genotype 1 strain, expressed in insect cells [126]. Two thousand soldiers in Nepal received three doses (20 µg) of this vaccine at month 0, 1 and 6. While this vaccine trial demonstrated promising results (induction of anti-HEV-Abs after the 3. Vaccination could be observed in 100%), further development of this vaccine was stopped. However, in 2010 another vaccine (HEV 239, Hecolin) passed successfully a phase 3 trial with more than 100,000 participants in China [40]. This vaccine also based on a protein encoded by ORF 2 of an HEV genotype 1. In the phase 3 study the long-term efficacy and safety of this vaccine was studied over more than four years in a vaccinated group of 56,302 participants in comparison with a control group of 56,302 participants. In this study, 60 cases of hepatitis E occurred. Only seven belonged to the vaccinated group, demonstrating the efficacy of this vaccine to be 87%. No relevant severe adverse events could be observed in context with the vaccine [40]. The protective effect of this vaccine could be proven for HEV genotype 1 and 4 infections, but nothing is known about genotype 3 infections. However, the NIH has started a phase 1 trial studying the safety of this vaccine and phase 2 and 3 trials will follow. For the first time the US FDA has approved a Chinese vaccine to enter a clinical trial in the United States. The upcoming results will demonstrate the use of this vaccine in an HEV genotype 3 region.

In the initial Chinese large Hecolin trial, pregnancy was an exclusion criterion, however, due to insecurity of presence of a pregnancy, 37 pregnant women inadvertently received the HEV 239 vaccine and no relevant severe adverse events occurred in these mothers or their babies leading to the interpretation that this vaccine seems to be safe in pregnant women [127].

## 9. Conclusions and Recommendations

The great majority of HEV infections take a self-limiting, asymptomatic clinical course. In addition, in the small subgroup of patients with overt hepatitis E, the majority of patients do not require any specific treatment. However, in immunosuppressed patients with chronic hepatitis E (genotype 3 or 4), pregnant women with acute liver failure (genotype 1/2, but also 4) or patients with risk factors of developing acute on chronic liver failure, there is a need for medicinal intervention. Unfortunately, there is no approved drug for the treatment of acute or chronic hepatitis E but ribavirin has been shown to be safe and efficient in small cohorts. Furthermore, ongoing or previous HEV infections have been associated with several assumed extrahepatic manifestations but a direct causal link has still not been proven.

## Figures and Tables

**Table 1:** Hepatitis E virus (HEV) genotypes.

HEV Genotype	Area	Sources of Infection *	Comment
GT 1	Tropical developing countries of Asia and Africa	Contaminated drinking water	No zoonotic relevance, no chronic infections described, fulminant courses in pregnant women with a mortality of up to 20% in the last trimester, association with pancreatitis but not with neurological symptoms assumed
GT 2	Tropical countries of Africa or Mexico/Central America	Contaminated drinking water	No zoonotic relevance, no chronic infections described
GT 3	Industrialized nations, worldwide distributed, autochthonous in Europe, North and South America, Australia, large parts of Asia	Foodborne zoonosis (mainly contact or consumption of inadequately cooked pork) shellfish deer Strawberries Vegetables (spinach, rocket) Blood transfusions	Chronic HEV infections described in several cohorts of immunosuppressed patients, frequently observed neurological symptoms (in particular Neuralgic amyotrophy) in association with HEV infections, no association with male infertility
GT 4	Mainly in Asia, recently single cases in Europe	Foodborne zoonosis (mainly contact or consumption of inadequately cooked pork) shellfishdeer	Chronic HEV infections described in single immunosuppressed patients, no clear evidence for an association with neurological symptoms, association with preterm birth and abortion, eventually associated with male infertility
GT 5	Japan	Wild boar	Relevance for humans still unclear
GT 6	Japan	Wild boar	Relevance for humans still unclear
GT 7	Middle East	Dromedary camels (one-humped camels)	Chronic infection in a liver transplant recipient who regularly consumed camel meat and milk
GT 8	Middle East	Bactrian camels (two-humped camels)	Relevance for humans still unclear
Rat hepatitis E	Hong Kong	Rats	Genetically distinct from the classical HEV genotypes and thus not classified regularly. Two known cases of human infections, one of them chronic in a transplant recipient
Avian hepatitis E	Worldwide	Birds	Genetically distinct from the classical HEV genotypes. No known human cases

GT, genotype. * vertical transmission of HEV has been observed [128,129].
